# Anti-Cancer Activity of *Solanum nigrum* (AESN) through Suppression of Mitochondrial Function and Epithelial-Mesenchymal Transition (EMT) in Breast Cancer Cells

**DOI:** 10.3390/molecules21050553

**Published:** 2016-04-28

**Authors:** Ying-Jang Lai, Chen-Jei Tai, Chia-Woei Wang, Chen-Yen Choong, Bao-Hong Lee, Yeu-Ching Shi, Cheng-Jeng Tai

**Affiliations:** 1 Department of Food Science, National Quemoy University, Quemoy 89250, Taiwan; d91641006@gmail.com; 2 Department of Chinese Medicine, Taipei University Hospital, Taipei 11042, Taiwan; chenjtai@tmu.edu.tw (C.-J.T.); f96b47117@ntu.edu.tw (B.-H.L.); 3 Traditioal Herbal Medicine Research Center, Taipei Medical University Hospital, Taipei 11042, Taiwan; 4 Department of Obstetrics and Gynecology, School of Medicine, College of Medicine, Taipei Medical University, Taipei 11042, Taiwan; cwwang@ms4.hinet.net (C.-W.W.); chenyen_318@hotmail.com (C.-Y.C.); 5 Department of Obstetrics and Gynecology, Taipei Medical University Hospital, Taipei 11042, Taiwan; 6 Division of Hematology and Oncology, Department of Internal Medicine, School of Medicine, College of Medicine, Taipei Medical University, Taipei 11042, Taiwan; 7 Division of Hematology and Oncology, Department of Internal Medicine, Taipei Medicine University Hospital, Taipei 11042, Taiwan

**Keywords:** epithelial-mesenchymal transition (EMT), aqueous extracts of *Solanum nigrum* (AESN), MCF-7 breast cancer cells, apoptosis, mitochondrial fission

## Abstract

Chemotherapy is the main approach for treating advanced and recurrent carcinoma, but the clinical performance of chemotherapy is limited by relatively low response rates, drug resistance, and adverse effects that severely affect the quality of life of patients. An association between epithelial-mesenchymal transition (EMT) and chemotherapy resistance has been investigated in recent studies. Our recent studies have found that the aqueous extract of *Solanum nigrum* (AESN) is a crucial ingredient in some traditional Chinese medicine formulas for treating various types of cancer patients and exhibits antitumor effects. We evaluated the suppression of EMT in MCF-7 breast cancer cells treated with AESN. The mitochondrial morphology was investigated using Mitotracker Deep-Red FM stain. Our results indicated that AESN markedly inhibited cell viability of MCF-7 breast cancer cells through apoptosis induction and cell cycle arrest mediated by activation of caspase-3 and production of reactive oxygen species. Furthermore, mitochondrial fission was observed in MCF-7 breast cancer cells treated with AESN. In addition to elevation of E-cadherin, downregulations of ZEB1, N-cadherin, and vimentin were found in AESN-treated MCF-7 breast cancer cells. These results suggested that AESN could inhibit EMT of MCF-7 breast cancer cells mediated by attenuation of mitochondrial function. AESN could be potentially beneficial in treating breast cancer cells, and may be of interest for future studies in developing integrative cancer therapy against proliferation, metastasis, and migration of breast cancer cells.

## 1. Introduction

The crude extracts of *Solanum nigrum* have demonstrated antitumor effects in various types of cancer including human melanoma and colorectal, endometrial, cervical, and breast cancers [1,2,3,4]. Recently, we have found that the aqueous extract of *Solanum nigrum* (AESN) could demonstrate anti-proliferation potential in various cancer cells [5,6,7,8]. Previous studies have indicated that AESN mainly suppressed tumor cell growth by apoptosis induction [5] and LC-3 A/B-related autophagy [5,6,7,8].

Epithelial-mesenchymal transition (EMT) is a process that results in invasive cells could enter the blood stream [9]. Studies have suggested that cancer stem cells undergo EMT to migration and invasion [10,11]. During EMT, a decrease was found in E-cadherin, occludins, claudins, and desmoplakin, as well as elevations of vimentin, N-cadherin, fibronectin, and alpha-smooth muscle actin) [12].

Genome-wide transcriptional analysis of human breast cancer cell lines has revealed a subgroup of cells with increased expression of EMT markers and high invasive potential, termed the mesenchymal type. These cells display a “mesenchymal” gene expression profile in contrast to a second subcategory, the luminal breast cancer cells, which exhibit poor invasive capability and low expression of EMT markers, and bear an epithelial gene expression profile [13,14].

## 2. Results

### 2.1. Suppression of MCF-7 Breast Cancer Cells through AESN Treatment

**Figure 1:**
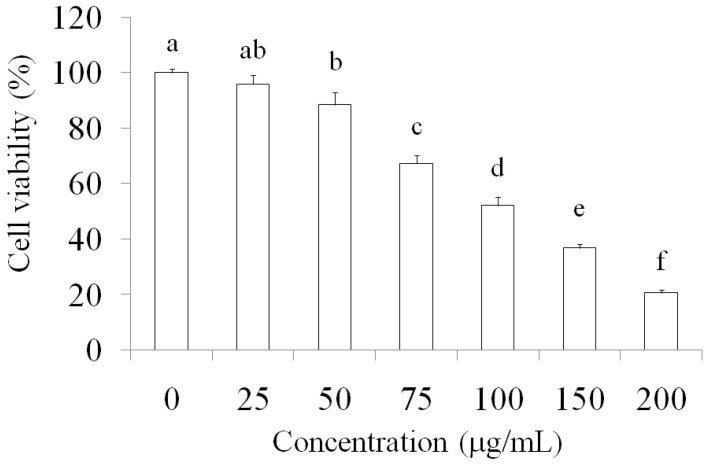
Cell viability of MCF-7 breast cancer cells treated by aqueous extract of *Solanum nigrum* (AESN) for 24 h. Data are shown as mean ± SD (*n* = 3). The significant differences were shown by different letters (*p* < 0.05).

**Figure 2:**
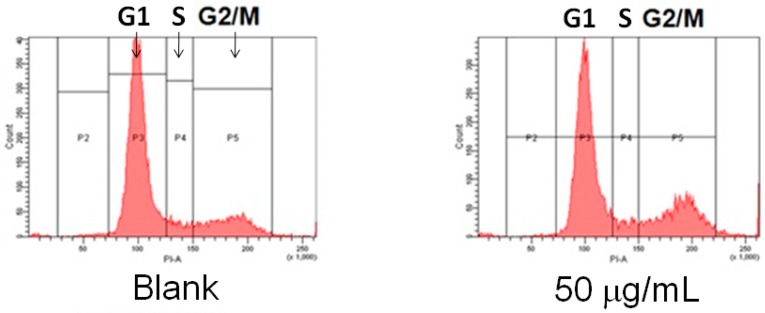

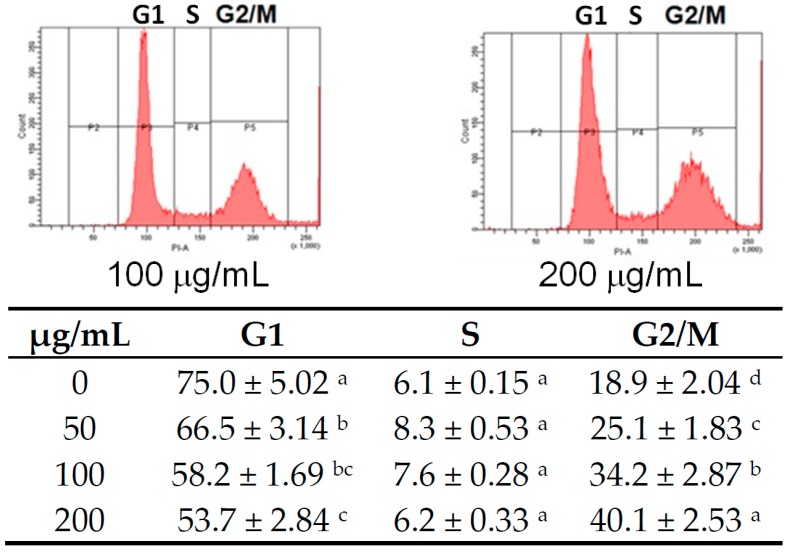
Cell cycle of MCF-7 breast cancer cells treated by AESN for 12 h. Data are shown as mean ± SD (*n* = 3). A significant difference is indicated by different letters in each column (G1, S, and G2/M phase) (*p* < 0.05).

After 24 h treatment by AESN, the cell viability of MCF-7 breast cancer cells was evaluated. As shown in Figure 1, the cell toxic effect of AESN on MCF-7 cells was appeared in a dosage-dependent manner. In addition, the cell cycle of MCF-7 cells was measured, and the results indicated that MCF-7 breast cancer cells were arrested in the G2/M phase after 12 h treatment with AESN (Figure 2). These results suggested that AESN could limit proliferation of MCF-7 breast cancer cells, resulting in cell death.

### 2.2. Apoptosis Induction by AESN Treatment in MCF-7 Breast Cancer Cells

**Figure 3:**
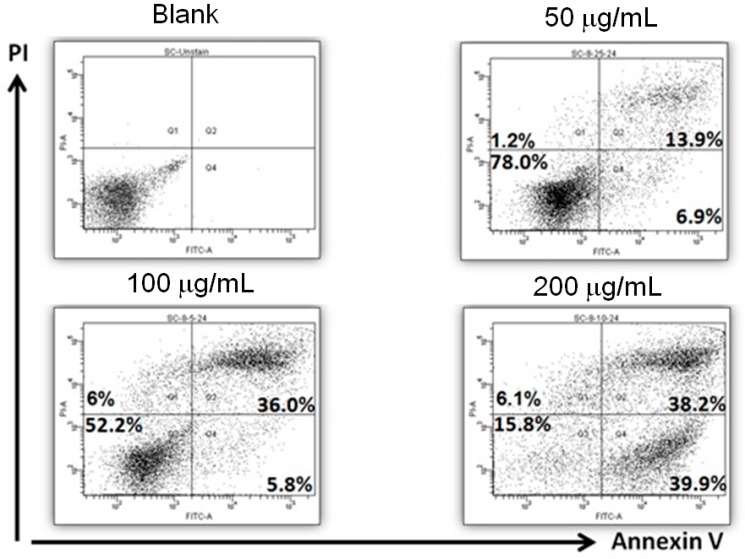
Measurement for apoptosis induction by AESN treatment (24 h) in MCF-7 breast cancer cells.

To confirm the potential of AESN-induced MCF-7 cells death, we investigated the apoptosis and necrosis of MCF-7 cells using the propidium iodide (PI)/Annexin-V double stain. This staining method combined with flow cytometry enables quantitatively assessing living (Annexin-V-FITC negative/PI negative), early apoptotic (Annexin-V-FITC positive/PI negative), late apoptotic/necrotic (Annexin-V-FITC positive/PI positive), and dead (Annexin-V-FITC negative/PI positive) cells. As shown in Figure 3, AESN clearly resulted in apoptosis in MCF-7 breast cancer cells after 24 h treatment.

### 2.3. Measurements of Caspase-3 and Reactive Oxygen Species (ROS) Level

**Figure 4:**
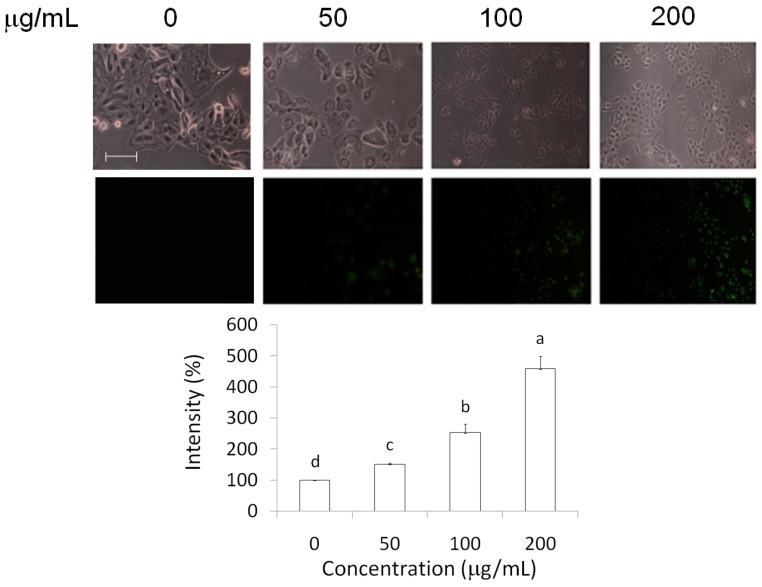
Activation of caspase-3 (FITC-conjugate secondary antibody) in AESN-treated MCF-7 breast cancer cells after 24 h treatment by fluorescent microscopy. Data are shown as mean ± SD (*n* = 3). A significant difference is shown by different letters (*p* <0.05). The fluorescent intensity was calculated and normalized according to cell number in each group. Scale bar: 100 μm.

**Figure 5:**
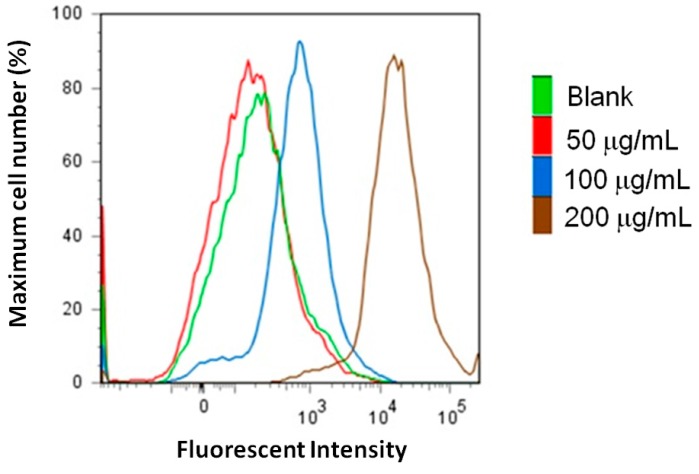
Reactive oxygen species (ROS) level of MCF-7 breast cancer cells treated by AESN for 24 h and stained by dichlorodihydrofluorescin diacetate (DCFH-DA).

Apoptosis has been found to be regulated by two main pathways, the mitochondrial pathway and the death receptor pathway, both of which activate caspase-3 [15]. Hence, we evaluated the elevation of caspase-3 levels in MCF-7 breast cancer cells treated with AESN. We found that AESN markedly increased the caspase-3 levels by fluorescent stain as shown in Figure 4. In addition, AESN clearly increased the reactive oxygen species (ROS) level in MCF-7 breast cancer cells according to dichlorodihydrofluorescin diacetate (DCFH-DA) stain after 24 h treatment (Figure 5).

### 2.4. Mitochondrial Morphology

**Figure 6:**
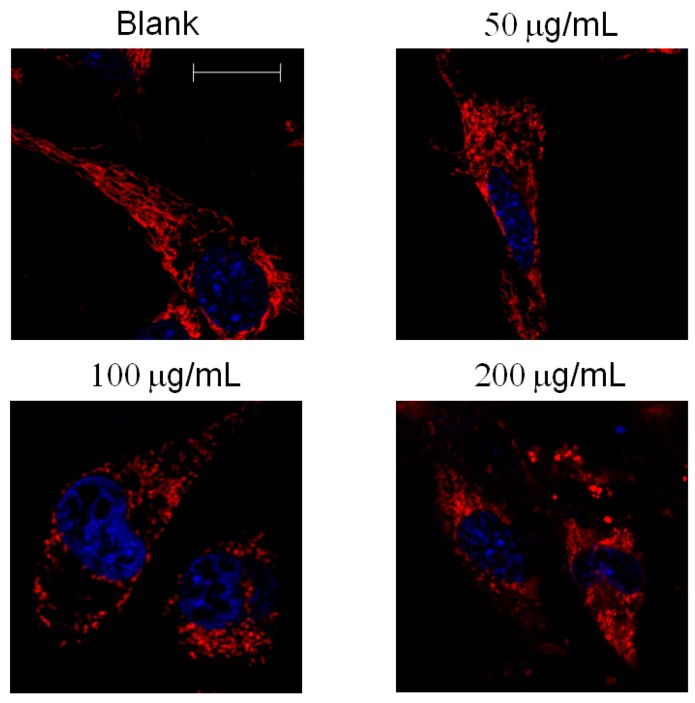
Observation of mitochondrial fission in MCF-7 breast cancer cells treated by AESN. Fission: mitochondrial fragment. Fusion: mitochondrial network. Scale bar: 20 μm.

Fission and fusion are important for growth, for mitochondrial redistribution, and for maintenance of a healthy mitochondrial network. In addition, mitochondrial fission and fusion play prominent roles in disease-related processes such as apoptosis and mitophagy. Mitochondrial fission can be stained according to a recent study [16]. We found that mitochondrial morphology exhibits clear change in MCF-7 breast cancer cells treated with AESN for 24 h. Mitochondria fission was induced and visualized using 100 μg/mL and 200 μg/mL of AESN treatment (Figure 6). This result indicated that AESN may affect mitochondrial activity and regulates proliferation and apoptosis in MCF-7 breast cancer cells.

### 2.5. EMT Marker

**Figure 7:**
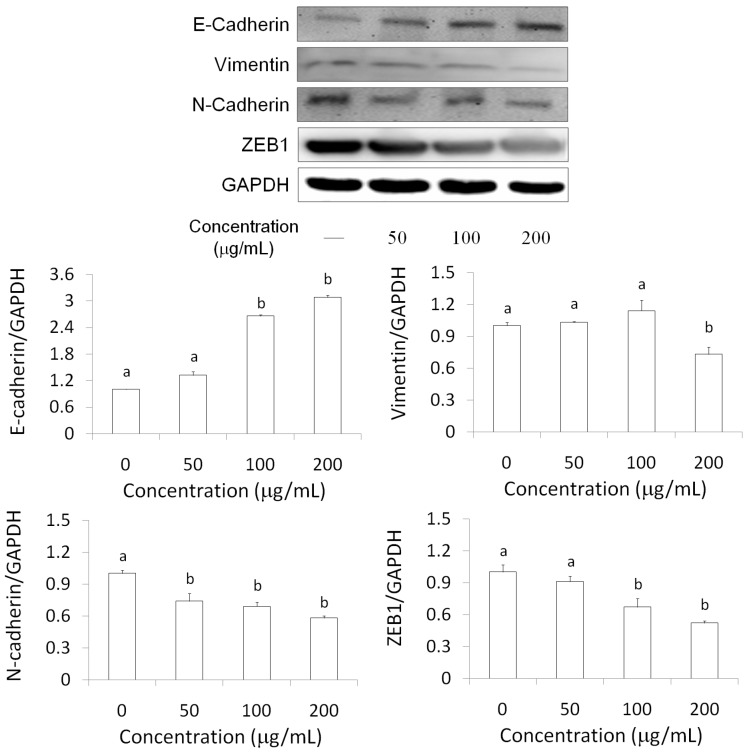
Regulations of AESN on E-cadherin, N-cadherin, vimentin, and ZEB1 in MCF-7 breast cancer cells. Data are shown as mean ± SD (*n* = 3). Significant difference is indicated by different letters (*p* < 0.05).

EMT is one of the main mechanisms in the development of cancer metastasis [17]. By undergoing EMT, cancer cells maximize their growth, migration, invasion, metastasis, and drug resistant abilities [18,19,20,21]. Therefore, a reversal of the EMT process is a potential therapeutic method for inhibiting metastasis and sensitizing cancer cells to chemotherapeutics [22]. We found that AESN (200 μg/mL) markedly attenuated N-cadherin, ZEB1, and vimentin expressions in MCF-7 breast cancer cells. In addition, the level of E-cadherin was elevated through AESN treatment for 24 h (Figure 7).

## 3. Discussion

With its wide applications in cancer prevention and treatment, the public interest in complementary and alternative medicine continues to increase worldwide. Traditional Chinese medicine is one of the most common and crucial applications in complementary and alternative medicine. Novel molecular prognostic markers, which have been shown to participate in specific pathways involved in the tumorigenesis and tumor progression of cervical cancer, may provide useful information for determining patient prognosis, predict survival, and design therapeutic strategies.

EMT is characterized by a morphological and functional shift from epithelial cells to fibroblast-like cells; this shift results in the loosening of intercellular junctions and increased cellular mobility [23,24]. E-cadherin is the major cell adhesion molecule that forms intracellular adhesion junctions in epithelial cells, and the loss of E-cadherin level has been suggested to be the first stage of cancer cell metastasis [25]. EMT has been demonstrated to play an essential role in cancer cell invasion and metastasis [26], and many different biomarkers involved in EMT have been identified, including E-cadherin, N-cadherin, fibronectin, and vimentin [26,27]. Previous results have suggested that reduced E-cadherin levels may enhance EMT and increase the migration of cancer cells [23]. In addition, a decreased in E-cadherin expression is associated with poor prognosis in cervical cancer patients [28,29]. Increased expression of EMT-related transcription factors, such as snail, slug, twist-2, and ZEB, has been shown to enhance chemotherapy resistance in human cervical cancer cells [30,31,32]. EMT-inducing factors activate different signals that finally converge in the expression of transcription factors that regulate EMT (families of Snail, ZEB, and Twist, among others). Snail (*Snai1* gene), which was proposed as an essential regulator of EMT during embryonic development, is a strong repressor of transcription of the E-cadherin gene [33]. MCF-7 cells was associated with increased expression of major transcription factors such as SNAIL, SLUG and ZEB-1, which are known to play a role in EMT [34]. Twist-2 has been described to be a direct repressor of E-cadherin *in vitro* and *in vivo* [35]. In this study, we found that AESN could attenuate N-cadherin, vimentin, and ZEB1 levels of MCF-7 breast cancer cells after 24 h treatment (Figure 7), revealing that AESN may demonstrate chemotherapy resistance, metastasis, and cancer cell migration as well as suppress cancer cell proliferation [1,5,6].

In addition to performing metabolic reactions, mitochondria also undergo fission/fusion changes, namely, mitochondrial dynamics, which plays a critical role in regulating cell metabolism, survival, and proliferation [36]. The molecular mechanisms that govern the fission/fusion dynamics have been partially illustrated. Fusion serves to unify the mitochondrial compartment, whereas fission generates morphologically and functionally distinct mitochondria. Mitochondrial fission often occurs early in the apoptotic event [37] and the autophagic process [38]. Fusion of mitochondria is associated with increased cell survival [39]. We found that AESN could change mitochondrial morphology and induced mitochondrial fission (Figure 6).

**Figure 8:**
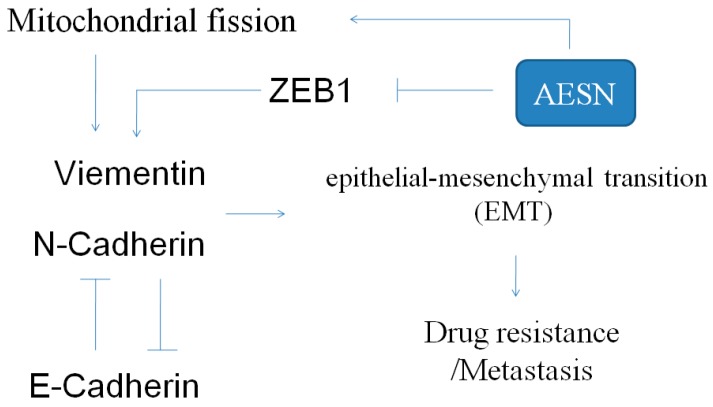
The potential mechanism through which AESN regulates EMT in MCF-7 breast cancer cells.

The aqueous extract of the *Solanum nigrum* leaf, a widely used medicinal herb in traditional Chinese medicine, demonstrated significant cytotoxicity inhuman breast cancer cells via suppression of EMT and apoptosis. Furthermore, it was also capable of enhancing mitochondrial fission, thereby attenuating mitochondrial function in the human breast cancer cell line (MCF-7 cells). These *in vitro* results suggested that the use of AESN could be potentially beneficial in treating breast cancer cells, and may be of interest for further studies in developing integrative cancer therapy against proliferation, metastasis, and migration of breast cancer cells (Figure 8).

## 4. Materials and Methods

### 4.1. Chemicals

The plant, *i.e.*, aqueous extracts of *Solanum nigrum* (AESN), was collected in Tainan, Taiwan. The *Solanum nigrum* (1 kg) was extracted with water (10 L) three times at room temperature. After evaporating the solvents under vacuum at 40 °C, a residue was obtained. Crystal violet, propidium iodide (PI), sodium dodecyl sulfate (SDS), Triton X-100, trypsin, and trypan blue were purchased from Sigma Chemical Co. (St. Louis, MO, USA). Fetal bovine serum (FBS) was purchased from Life Technologies (Auckland, New Zealand). Dimethyl sulfoxide was purchased from Wako Pure Chemical Industries (Saitama, Japan). Mitotracker Deep-Red FM was purchased from Invitrogen (Carlsbad, CA, USA). An anti-vimentin antibody, anti-caspase-3 antibody, anti-E-cadherin antibody, and anti-N-cadherin antibody were purchased from Santa Cruz (Santa Cruz, CA, USA).

### 4.2. Cell Culture

MCF-7 breast cancer cells (Bioresource Collection and Research Center, Hsinchu, Taiwan) were incubated with Eagle minimum essential medium with 2 mM l-glutamine, 1.5 g/L of sodium bicarbonate, 0.1 mM nonessential amino acids, 1.0 mM sodium pyruvate, and 10% FBS in 5% CO_2_ at 37 °C.

### 4.3. Cell Viablitiy

The cell-killing effect of AESN against breast cancer cells was measured using a crystal violet staining assay. Cells were seeded on 24-well plates (3 × 10^4^ cells per well) and treated with various concentrations of AESN for 24 h. The medium was then removed, washed with phosphate-buffered saline (PBS), and stained with 2 g/L of crystal violet solved in phosphate-buffered formaldehyde for 20 min before being washed with water. The crystal violet bound to the cells was dissolved in 20 g/L of SDS solution, and its absorbance at 600 nm was measured [14].

### 4.4. Cell Cycle

After treatment with AESN, the medium was aspirated and adherent cells were harvested and centrifuged at 300× *g* for 5 min. Cells were washed with PBS, fixed with ice-cold ethanol at −20 °C overnight, and then stained with PI at room temperature for 30 min. The cell cycle distribution was analyzed using flow cytometry using a FACScan-LSR flow cytometer equipped with CellQuest software (Version 3.3, BD Biosciences, San Jose, CA, USA).

### 4.5. Apoptosis

For apoptosis detection, floating cells in the medium and adherent cells were collected after AESN treatment. Cells were harvested, washed in ice-cold PBS, and resuspended in 200 μL of binding buffer before being incubated in 5 μL of AnnexinV-FITC (BD Biosciences) solution and 5 μL of PI at room temperature for 15 min in the dark. Subsequently, 300 μL of binding buffer was added. Cells were analyzed using flow cytometry. Untreated cells were used as the control for double staining.

### 4.6. Western Blot

Cells were rinsed with ice-cold PBS and lysed by RIPA lysis buffer with protease and phosphatase inhibitors for 20 min on ice. Then, the cells were centrifuged at 12,000× *g* for 10 min at 4 °C. Protein extracts (20 μg) were resolved using SDS-polyacrylamide gel electrophoresis (SDS-PAGE; 200 V, 45 min). The protein bands were electrotransferred to nitrocellulose membranes (80 V, 120 min). Membranes were then treated with a 5% enhanced chemiluminescence (ECL) blocking agent (GE Healthcare Bio-Sciences) in saline buffer (T-TBS) containing 0.1% Tween-20, 10 mM Tris-HCl, 150 mM NaCl, 1 mM CaCl_2_, and 1 mM MgCl_2_ at a pH of 7.4 for 1 h, and then incubated with the primary antibody overnight at 4 °C. Subsequently, membranes were washed three times in T-TBS, and proteins were detected using appropriate horseradish peroxidase-conjugated secondary antibodies, followed by analysis in an ECL plus Western blotting detection system (GE Healthcare Bio-Science).

### 4.7. Immune Fluorescent Stain

Cells were permeabilized and fixed by PBS (contained 0.1% Triton X-100) for 5 min at room temperature. The cell slides were then washed three times in PBS for 5 min at room temperature. After blocking, the slides were laid flat in a humidified chamber and incubated for 1–2 h at room temperature. One hundred microliters of the caspase-3 antibody (1:200) were added, and slides were incubated in a humidified chamber overnight at 4 °C. After being washed with PBS, 100 μL of a FITC-conjugate secondary antibody was dissolved in PBS (1:500) and incubated for 2 h at room temperature. The fluorescent intensity of caspase-3 was observed using a fluorescence microscope.

### 4.8. ROS Production

Cells were suspended in 500 μL of PBS, and mixed with 10 μM (final concentration) DCFH-DA and incubated for 10 min at 37 °C. The reactive oxygen species (ROS) level was assayed using flowcytometry (Becton–Dickinson, San Jose, CA, USA) [15].

### 4.9. Mitochondrial Fission

Cells were treated with 250 nM Mitotracker Deep-Red FM (Invitrogen) for 30 min in a serum-free culture medium. After being washed with PBC twice, nuclei were stained with Hochest 33342 for 10 min. The mitochondrial morphology was observed using a confocal microscope [16].

### 4.10. Statistical Analysis

The analysis of variance was used to evaluate the significance of the differences between factors and levels. Comparison of the means was carried out by employing a Student’s *t*-test to identify which groups were significantly different from other groups. The least significant difference was *p* < 0.05.

## 5. Conclusions

The aqueous extract of the *Solanum nigrum* leaf, a widely used medicinal herb in traditional Chinese medicine, demonstrated significant cytotoxicity in human breast cancer cells via suppression of EMT and apoptosis. Furthermore, it was also capable of enhancing mitochondrial fission, thereby attenuating mitochondrial function in the human breast cancer cells. These *in vitro* results therefore suggest that the use of AESN could be potentially beneficial in treating breast cancer cells, and may be of interest for further studies in developing integrative cancer therapy against proliferation, metastasis, and migration of breast cancer cells.

## Abbreviations

The following abbreviations are used in this manuscript:

AESN: aqueous extract of *Solanum nigrum*
DCFH-DA: dichlorodihydrofluorescin diacetate
EMT: epithelial-mesenchymal transition
FBS: fetal bovine serum
PBS: phosphate-buffered saline
PI: propidium iodide
ROS: reactive oxygen species
SDS: sodium dodecyl sulfate
